# Next-generation cell-penetrating antibodies for tumor targeting and RAD51 inhibition

**DOI:** 10.18632/oncotarget.28651

**Published:** 2024-10-01

**Authors:** Madison Rackear, Elias Quijano, Zaira Ianniello, Daniel A. Colón-Ríos, Adam Krysztofiak, Rashed Abdullah, Yanfeng Liu, Faye A. Rogers, Dale L. Ludwig, Rohini Dwivedi, Franziska Bleichert, Peter M. Glazer

**Affiliations:** ^1^Department of Therapeutic Radiology, Yale University School of Medicine, New Haven, CT 06520, USA; ^2^Department of Genetics, Yale University School of Medicine, New Haven, CT 06520, USA; ^3^Gennao Bio, Hopewell, NJ 08525, USA; ^4^Department of Molecular Biophysics and Biochemistry, Yale University, New Haven, CT 06520, USA

**Keywords:** 3E10, cell penetration, nucleic acid binding, nucleic acid delivery, RAD51

## Abstract

Monoclonal antibody therapies for cancer have demonstrated extraordinary clinical success in recent years. However, these strategies are thus far mostly limited to specific cell surface antigens, even though many disease targets are found intracellularly. Here we report studies on the humanization of a full-length, nucleic acid binding, monoclonal lupus-derived autoantibody, 3E10, which exhibits a novel mechanism of cell penetration and tumor specific targeting. Comparing humanized variants of 3E10, we demonstrate that cell uptake depends on the nucleoside transporter ENT2, and that faster cell uptake and superior *in vivo* tumor targeting are associated with higher affinity nucleic acid binding. We show that one human variant retains the ability of the parental 3E10 to bind RAD51, serving as a synthetically lethal inhibitor of homology-directed repair *in vitro*. These results provide the basis for the rational design of a novel antibody platform for therapeutic tumor targeting with high specificity following systemic administration.

## INTRODUCTION

In past decades, monoclonal antibody (mAb) therapy for cancer has emerged as a powerful treatment against many hematologic and solid malignancies, with 40 therapies currently approved by the FDA [1]. Unlike conventional chemotherapy, mAbs, which bind specific epitopes, can provide precise tissue, cell, or tumor targeting as well as anti-tumor immune activation, thus providing an alternative treatment strategy to drugs with high systemic toxicity. Examples include checkpoint immune therapies like pembrolizumab and tremelimumab [2, 3], as well as classes of antibodies that target mutant or overexpressed cell surface receptors like HER2 [4] and EGFR [5]. Furthermore, refined conjugation methods have linked antibodies to small-molecule drugs (antibody-drug conjugates) [6], lipid nanoparticles [7, 8], and, most recently, proteolysis targeting chimeras [9, 10]. Although many mAbs were originally discovered or generated in mice, most are now humanized during commercialization to avoid human anti-mouse antibody responses that can cause allergic reactions, increase the rate of mAb clearance, and decrease mAb penetration into the tumor [11].

While antibody therapies have thus far led to profound improvements in clinical outcomes, intracellular delivery remains challenging. Despite the fact that as many as two-thirds of all disease-associated targets are localized inside the cell [12], there are no effective strategies for cytosolic delivery of mAbs that can avoid lysosomal degradation. To bypass this issue, two alternative modalities of high interest are cell-penetrating peptides [13–15] and anti-nuclear or anti-DNA antibodies, often derived from systemic lupus erythematosus (SLE) [16, 17]. Given their size, both cell-penetrating peptides and anti-DNA mAbs are generally required to enter cells via receptor-mediated endocytosis (e.g., target receptors for cell-penetrating peptides and mAbs, and Fc receptors and neonatal Fc receptors for IgG antibodies, which comprise the largest class of clinically available therapeutic mAbs). The only means of avoiding eventual lysosomal degradation is endosomal escape, which occurs at low frequencies, and even then may lead to cytosolic degradation [18]. There are, however, two notable well-studied anti-DNA, SLE-derived antibodies that bypass the endocytic pathway: 3D8, which enters cells via caveosomes and efficiently escapes into the cytosol prior to endosomal trafficking [19], and 3E10, which is the focus of this work.

3E10 is a unique anti-DNA antibody with many interesting properties. Notably, 3E10 specifically localizes to tumors following systemic intravenous injection [20, 21], penetrates into tumor cells [22], and delivers non-covalently bound nucleic acids into cells *in vivo* [23]. Though the full internalization mechanism has yet to be elucidated, prior work has robustly demonstrated a distinct dependence on the nucleoside transporter ENT2 [20, 22], which is present in both plasma and nuclear membranes [24, 25]. ENT2 is also overexpressed in many human malignancies, including gynecologic and liver cancers [26–28], prompting its interest as a therapeutic target [29]. 3E10 cell penetration is dependent on the presence of extracellular DNA [21] as well as the ability to bind DNA [30], which are important properties for tumor localization, as extracellular DNA tends to accumulate in necrotic tumors [31] and is correlated with poor patient survival [32]. Finally, we previously reported that 3E10 binds to the DNA repair protein RAD51, inhibiting homology-directed repair (HDR) [30], and is consequently synthetically lethal to cells with dysfunctional DNA repair, including BRCA2-deficient and PTEN-deficient cells [21, 30, 33].

Here we report findings from studies of humanized versions of full-length 3E10, which we engineered to avoid the emergence of human anti-mouse antibody responses which were previously seen in a small cohort of patients who received the murine 3E10 antibody in a Phase I clinical trial [34]. Previous attempts to humanize 3E10 have focused on divalent single-chain variable fragments (scFvs) and have been limited in scope, evaluating only synthetic lethality as opposed to the important nucleic acid binding, nucleic acid delivery, and direct RAD51 binding properties [35]. We therefore sought to screen full-length humanized versions of 3E10 and evaluate the known properties of the antibody in order to optimize it for multiple types of payload delivery and intracellular functions in the pursuit of clinical development.

We performed complementarity-determining region (CDR) grafting and mutated predicted important variable heavy and light chain residues to generate a total of 22 full-length humanized 3E10 variants in an IgG1 framework. Identifying three variants of high, medium, and low nucleic acid affinity, we use *in silico* structural prediction and electrostatic modeling to identify a nucleic acid binding pocket whose charge determines nucleic acid binding affinity. We find that humanized 3E10 cell penetration is dependent on an ENT2-related mechanism and that higher affinity nucleic acid binding is correlated with more robust uptake, enhanced tumor targeting, slower intracellular release of an mRNA ligand, and decreased RAD51 binding. Taken together, these results provide the basis for a novel, rationally engineered, antibody-based approach for potent intratumoral delivery for multiple applications in clinical oncology.

## RESULTS

### Generation of multiple humanized 3E10 antibody variants produces a wide variety of antibody nucleic acid affinities

**Figure 1:**
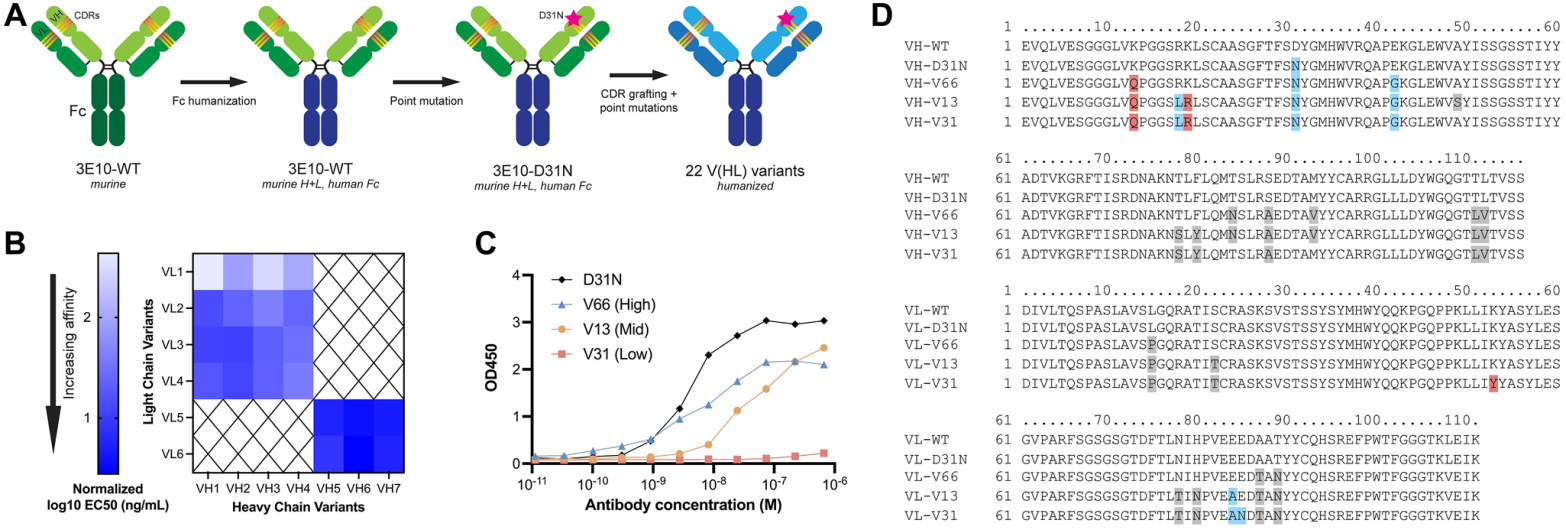
3E10 humanization and heavy and light chain variant screening. (**A**) Diagram of the 3E10 antibody engineering process. The original murine wild-type 3E10 was modified to contain a human IgG1 Fc region. This chimera was subsequently engineered with a D31N mutation in heavy chain CDR1. CDR grafting was performed to produce a fully humanized IgG1 framework; 22 variants were created by introducing point mutations into the VH and VL regions outside of the CDRs. (**B**) Nucleic acid affinity screening of humanized 3E10 variants. The 22 full-length antibodies were screened for their affinity to a 20-mer poly(dT) DNA oligo by ELISA for EC_50_ determination. All EC_50_s presented in the heat map are normalized to a chimeric 3E10 D31N positive control. (**C**) Representative ELISA assay data for poly(dT) binding by humanized V66, V13, and V31, and chimeric D31N. One biological replicate was performed. Humanized variant EC_50_s are 5.933, 44.34, and 685.2 nM, respectively. (**D**) Variable heavy chain (top) and variable light chain (bottom) sequence alignments, with deviations from WT sequence highlighted in red if substitution is increasingly anionic, blue if substitution is increasingly cationic, and grey if no significant change in formal charge at physiological pH.

The anti-DNA antibody 3E10 was originally discovered and isolated in a mouse model of SLE, and later was shown to be cell-penetrating with nuclear localization abilities [36, 37]. More recently, 3E10 was found to bind RAD51 and to be synthetically lethal in BRCA2- and PTEN-deficient cells [33], and new pre-clinical studies have also demonstrated the ability of 3E10 to deliver nucleic acids into tumor cells (with functional release) following systemic administration [23]. It is known that a substitution of asparagine in place of aspartic acid at position 31 in heavy chain CDR1 leads to higher affinity DNA binding and more robust cellular penetration [30]. Using this information, we sought to engineer humanized next generation 3E10 antibodies with tunable nucleic acid binding and release, cellular uptake, and RAD51 binding for various clinical applications. We first engineered full-length chimeric WT and D31N 3E10, which contain the original murine variable chains with a human IgG1 Fc domain. To overcome the drawbacks of murine antibody therapeutics for clinical use [38], we further sought to fully humanize 3E10. We identified potential key residues using two human germlines with high mouse homology, and from these generated 7 heavy chain and 6 light chain sequences. We used CDR grafting to create 22 permutations of fully humanized 3E10 variants, each containing the D31N mutation, in an IgG1 framework (Figure 1A). For ease of identification, we chose a nomenclature system wherein humanized variants are named according to their ordinal heavy and light chain sequences, i.e. variant VH1 + VL1 is deemed V11, variant VH1 + VL2 is deemed V12, and so forth. We screened all 22 variants for their affinity for poly(dT) DNA oligos by ELISA and found that the humanized 3E10 antibodies exhibited extremely variable EC_50_s spanning multiple orders of magnitude (Figure 1B and Supplementary Figure 1). We then chose one high affinity (V66, EC_50_ = 5.933 nM), one medium affinity (V13, EC_50_ = 44.34 nM), and one low affinity variant (V31, EC_50_ = 685.2 nM) for downstream characterization (Figure 1C). Hypothesizing that point mutational differences are responsible for the drastic changes in nucleic acid affinities, we aligned the heavy and light chain variable regions of WT and D31N chimeric 3E10 and V66, V13, and V31 (Figure 1D). In addition to the previously established critical nucleic acid binding residue at heavy chain position 31, we also noted additional residues that we hypothesized might affect nucleic acid affinity, including light chain residue K53, which is mutated only in the lowest affinity V31 variant.

### Nucleic acid binding pocket electrostatic charge predicts 3E10 nucleic acid affinity, intracellular payload localization, and release kinetics

**Figure 2:**
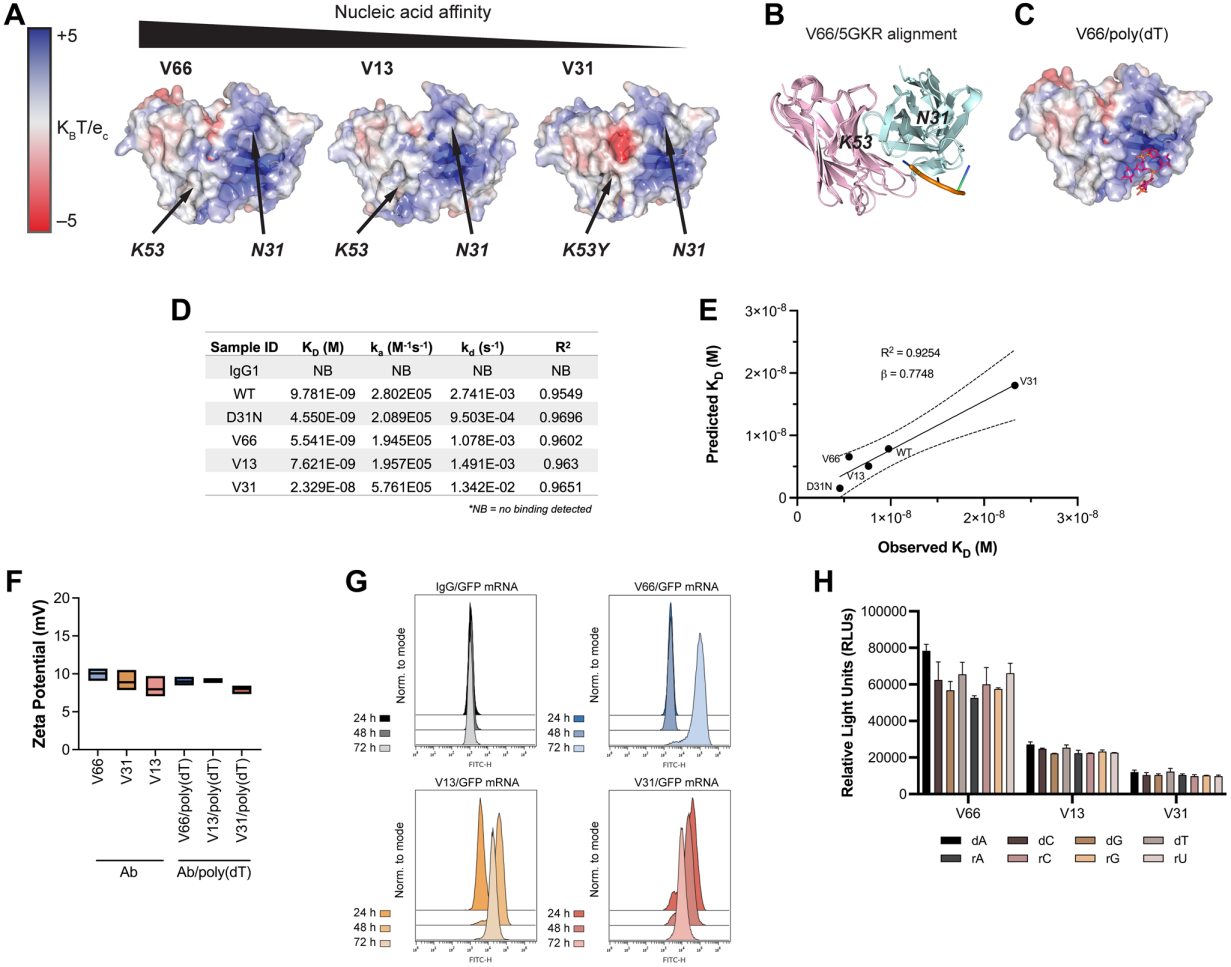
*In silico* modeling and *in vitro* characterization of 3E10 humanized variants reveal correlations between nucleic acid affinity and intracellular payload delivery. (**A**) *In silico* electrostatic modeling of V66, V13, and V31 using IgFold. Positions of N31 and K53 are labeled. (**B**) The crystal structure of the SLE anti-DNA antibody 5GKR overlaid with V66. Light chains are shown in pink and heavy chains are shown in teal. 5GKR was co-crystallized with 4-mer poly(dT), shown in orange. (**C**) Predicted binding of V66 and 4-mer poly(dT) modeled using AutoDock Vina. (**D**) Biolayer interferometry (BLI) assay to determine antibody equilibrium dissociation constants (K_D_). 20-mer biotinylated poly(dT) was adhered to a streptavidin chip. Antibody on-rates (k_a_) were measured for 5 min and off-rates (k_d_) were measured for 10 min. One biological replicate was performed. (**E**) Observed K_D_ values from BLI plotted against predicted K_D_ values calculated using AutoDock Vina. Plot shows best-fit simple linear regression (solid line) with 95% CI (dashed lines). Slope (β) and coefficient of determination (R^2^) are reported. (**F**) Assay for surface zeta potential of 3E10 human antibodies alone and antibody complexes with poly(dT), *n* = 3 replicates. Box plots represent upper and lower values with line at mean. (**G**) Representative flow cytometry traces of GFP mRNA expression at 24, 48, and 72 h. K562 cells were treated with 250 μg V66, V13, or V31 complexed with 10 μg mRNA encoding GFP. An IgG1 isotype control/GFP mRNA mixture was used as a negative control. 3 biological replicates were performed, and one representative result is shown. (**H**) ELISA assay for 3E10 antibody binding to mononucleotide DNA and RNA oligomers. Data are mean ± SEM, *n* = 3 replicates.

To explore which if any of these residues are critical for nucleic acid ligand binding, we used IgFold [39], an open-source deep learning tool, to predict the structures of 3E10 variant scFvs and model their surface electrostatic potentials. In accordance with previous work [30], we find a cationic pocket in a region of the antibody flanked by N31 and K53 (Figure 2A). Our hypothesis that nucleic acids bind in this pocket was strengthened by the fact that mutations in each of these residues (i.e., D31 in WT, K53Y in V31) are associated with greatly altered nucleic acid affinity. We find that in general, as antibody variant affinity decreases, an anionic pocket between N31 and K53 becomes more exposed; given the anionic nature of nucleic acids, this again reinforced our hypothesis that we had identified 3E10’s relevant ligand binding region.

To corroborate this hypothesis, we overlaid our high affinity (V66) structural model with the known crystal structure of 5GKR, another SLE anti-DNA antibody which was co-crystalized with a 4-mer poly(dT) ssDNA oligo [40] (Figure 2B). We furthermore used AutoDock Vina [41] to predict the most likely binding region of V66 and the same 4-mer poly(dT) (Figure 2C). We firstly find high structural homology between 3E10 (V66) and 5GKR, and secondly that the nucleic acid binding residues which we had predicted for 3E10 based on electrostatics are in the same structural region as the binding site on 5GKR.

We next sought to experimentally characterize the stability of noncovalent 3E10/nucleic acid complexes. For a more precise quantitative measure of 3E10/poly(dT) affinities, and to verify the accuracy of AutoDock Vina for such predictions, we performed biolayer interferometry (BLI). Notably, we observe that while the equilibrium dissociation constants (K_D_) of all variants are of similar low-nanomolar values (apart from the low-affinity V31), the off-rates (k_d_) vary more between WT and D31N, as well as between V31 and the other humanized variants (Figure 2D). This interesting finding suggested to us that the ligand dissociation kinetics of 3E10 variants may lead to variable intracellular nucleic acid delivery rates. We also find that the predicted equilibrium dissociation constants calculated using AutoDock Vina correlate with the observed BLI values (R^2^ = 0.9254) and furthermore that these values strongly quantitatively agree (β = 0.7748, Figure 2E). Finally, to test complex stability, we analyzed the surface zeta potential of 3E10 antibodies alone and 3E10/poly(dT) complexes. We find that for all antibodies and antibody/DNA complexes, the average zeta potential lies in the range of 7.5–10 mV (Figure 2F). Since zeta potential is not significantly altered with the addition of the DNA ligand, we conclude that addition of the ligand does not destabilize the 3E10 antibody in solution.

Work testing chimeric D31N to deliver nucleic acids into tumor cells has suggested the utility of 3E10 as an intracellular nucleic acid delivery vehicle for therapeutic use in solid tumors [23]. In our humanized variants, the differences in observed off-rates as measured by BLI led us to hypothesize that these variants may exhibit significantly different nucleic acid payload release kinetics following cellular internalization. To interrogate this hypothesis, we first confirmed that the trend for poly(dT) binding affinities of humanized variants was retained for another nucleic acid ligand, a GFP-encoding mRNA (Supplementary Figure 2). We then tested antibody-mediated delivery of GFP mRNA into cells using noncovalently formed antibody/GFP mRNA complexes. To do so, we followed GFP expression in cells treated with complexes continuously over the course of 3 days. We find that GFP is robustly expressed in cells in culture following treatment with all 3E10 antibody/mRNA complexes, as assayed by flow cytometry (Figure 2G). However, we noted that the time between antibody/GFP mRNA cell treatment and maximal GFP expression varies. We first observe GFP expression at 24 h for V31, 48 h for V13, and 72 h for V66. These differences correlate well with the K_D_ and k_d_ values for each antibody variant. The lowest affinity variant, V31, shows the most rapid GFP expression, suggesting the fastest release of the mRNA cargo. This is consistent with V31 having the highest k_d_ constant as measured by BLI. The opposite is true for the highest affinity variant, V66. Taken together, these data demonstrate that while all humanized variants tested here exhibit potent functional mRNA delivery *in vitro*, their nucleic acid release kinetics are variable and are related to their respective affinities.

We next tested whether the different 3E10 variants may exhibit differences in nucleic acid sequence specificity, leading to more efficient binding of certain ligands with certain variants that cannot be predicted by affinity studies with any other ligand. This hypothesis was based on an early report suggesting that the original murine 3E10 had a binding preference for poly(dT) over d(A), d(C), and d(G) [37]. However, this publication utilized a sub-optimal competitive binding assay, leading to conclusions we now believe were spurious, and which motivated us to revisit this question. To do so, we performed sensitive ELISA binding assays using deoxyribose- or ribose-based oligomers, each containing a single nucleotide species. We do not observe significant preferential sequence binding for any humanized 3E10 variant (Figure 2H), and we notably observe robust binding to all oligonucleotides tested.

### Variant nucleic acid affinity predicts cellular and tumor penetration, and variant delivery is associated with dependency on the human nucleoside transporter ENT2

**Figure 3:**
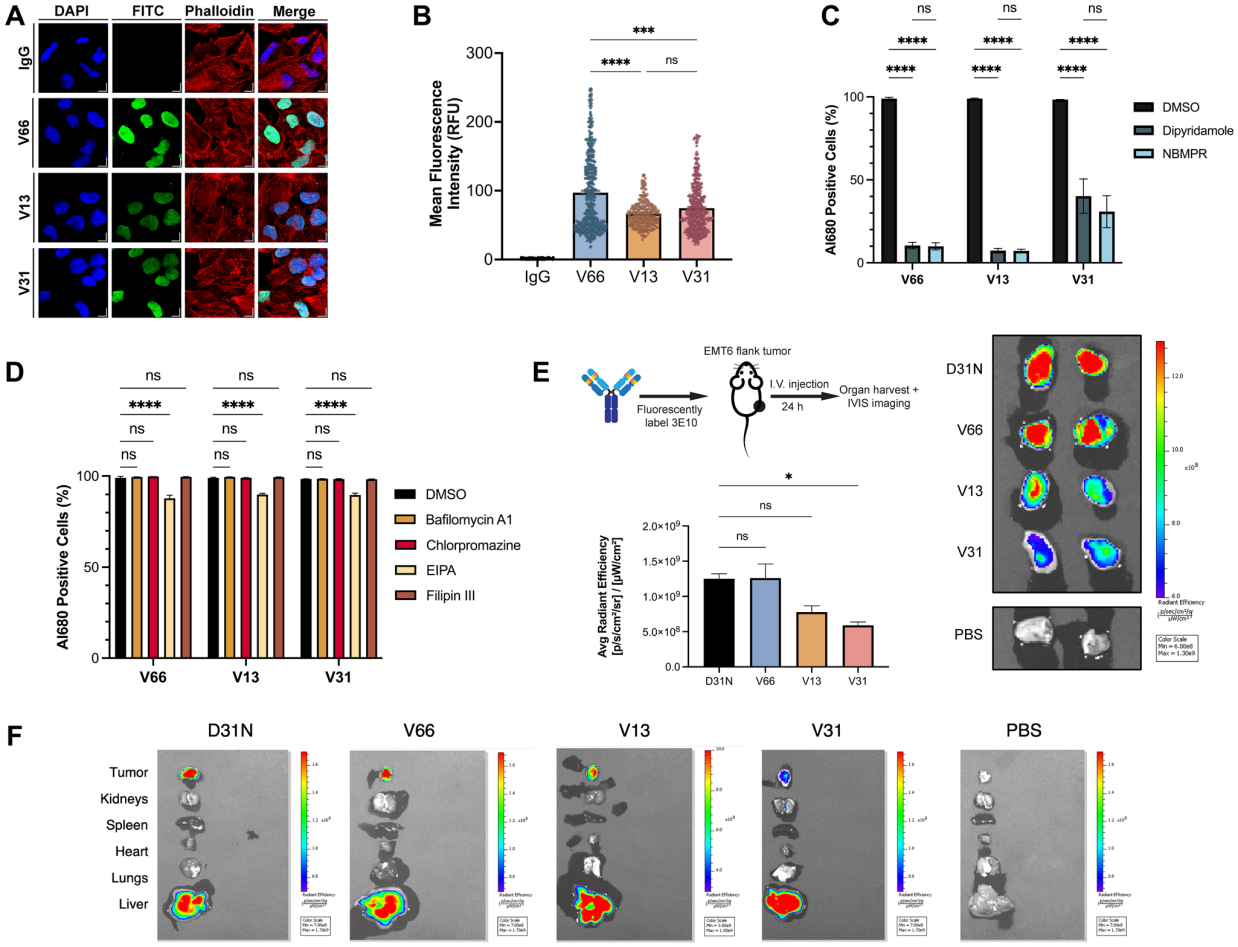
Characterization of 3E10 cellular penetration, mechanism of uptake, and tumor targeting. (**A**) Representative confocal immunofluorescence images and of HeLa cells treated with 750 nM humanized AlexaFluor 680-labeled 3E10 variants for 24 h. (**B**) Quantification of (A). *n* ≥ 250 cells per treatment group. (**C**, **D**) Quantification of antibody uptake in K562 cells treated with inhibitors of ENT2 (C) and of canonical cellular uptake pathways (D) as assessed by flow cytometry. Cells were treated with 750 nM AlexaFluor 680-labeled antibody for 2 h. Labeled IgG1 isotype was used as a negative control. Al680: AlexaFluor 680; EIPA: 5-(N-ethyl-N-isopropyl)-amiloride; NBMPR: S-(4-nitrobenzyl)-6-thioinosine. Data are mean ± SEM, *n* = 3 replicates. (**E**) Quantification and representative IVIS images of AlexaFluor 680 fluorescence in EMT6 mouse tumors isolated from tumor-bearing mice. Mice (*n* = 2 per group) were treated intravenously with AlexaFluor 680-labeled antibody (100 μg) and tumors were harvested 24 h after treatment. (**F**) Representative IVIS images showing fluorescence signal in mice injected intravenously with AlexaFluor680-labeled antibodies. One representative image of all major organs plus EMT6 tumors is shown per treatment group.

Multiple prior studies using the original murine version of 3E10 have robustly demonstrated its ability to penetrate cells in culture [21, 36, 42–44]. It has previously been shown, using the WT and D31N chimeric versions of 3E10, that cellular penetration increases as nucleic acid binding affinity increases [30]. In addition, prior published data has demonstrated the essential function of the ENT2 nucleoside transporter for 3E10 uptake [22]. Understanding these properties in the context of our humanized variants is important for eventual applications of 3E10 in the clinic, and we therefore asked whether each of these findings would hold true for the newly humanized variants. We first assessed general antibody uptake to confirm the cellular penetration abilities suggested by our mRNA delivery experiment. We assayed antibody cellular internalization after 24 h continuous treatment using confocal immunofluorescence microscopy (Figure 3A, 3B). We observe a significantly higher mean fluorescence intensity for V66 than for V13 and V31 corresponding to higher intracellular accumulation of V66. The increased uptake of the highest affinity humanized variant suggests that differences in nucleic acid affinity are correlated with cellular penetration ability. Notably, all variants do still show evidence of cellular and furthermore of nuclear penetration, demonstrating that this important and unique biological property is retained through the humanization process. This corroborates prior data, confirming both that nucleic acid binding facilitates 3E10 cellular penetration and that higher affinity binding leads to more robust uptake.

The previous finding that 3E10 uptake is dependent on the ENT2 transporter is clinically interesting for many reasons, including that transduction via ENT2 rather than canonical receptor-mediated endocytosis can lead to increased cytosolic availability and effector function of the antibody by avoiding lysosomal degradation [18]. We therefore investigated whether cellular uptake of 3E10 humanized variants is dependent upon ENT2 or other common membrane trafficking pathways. To do so, we treated cells in culture with fluorescently labeled humanized antibodies and analyzed antibody internalization via flow cytometry. We used two known chemical inhibitors of ENT2, dipyridamole and *S*-(4-nitrobenzyl)-6-thioinosine, which have previously been shown to inhibit 3E10 cellular uptake [20, 22]. We observe that, in accordance with previous findings, both inhibitors lead to a significant decrease in antibody uptake for all humanized variants (Figure 3C). We also confirmed that all cell lines used for 3E10 and 3E10/nucleic acid intracellular delivery studies in this publication are positive for ENT2 expression as assessed by western blotting (Supplementary Figure 3). Interestingly, we see that V66 and V13 uptake are each approximately 90% inhibited following cell treatment with ENT2 inhibitors, while V31 penetration is reduced by approximately 65%. While the exact role of ENT2 in 3E10 membrane transduction is an area of ongoing research, this finding suggests that nucleic acid binding may also play a role in the interaction with and internalization of 3E10 via ENT2, and that there may be compensatory or alternate uptake pathways that are utilized by 3E10 variants to different extents.

To understand other mechanisms which might be driving cellular penetration, we also treated cells with chemicals known to inhibit autophagy, macropinocytosis, clathrin-mediated endocytosis, and caveolae-mediated endocytosis. We find firstly that no inhibitors of autophagy (bafilomycin A1) or endocytic pathways (chlorpromazine for clathrin-mediated endocytosis or filipin III for caveolae-mediated endocytosis) affect the uptake of any humanized variants (Figure 3D). We also see that compared to ENT2 inhibitors, 5-(N-ethyl-N-isopropyl)-amiloride, an amiloride known to inhibit macropinocytosis, leads to a small but detectable reduction in 3E10 uptake of approximately 10%. Notably, cellular uptake of the anti-DNA antibody 2C10 in macrophages is thought to occur via macropinocytosis [16].

Prior work has also shown that chimeric D31N 3E10 localizes to murine tumors *in vivo* following systemic administration via intravenous injection [20, 21, 23]. Given that this is a critical property of the antibody for its eventual therapeutic development, we asked whether this biodistribution pattern held true for our three humanized variants of interest. To do so, we treated BALB/c mice bearing syngeneic EMT6 murine breast tumors (implanted subcutaneously in the flanks) with fluorescently labeled humanized 3E10 variants using systemic intravenous injections, and then used IVIS imaging to determine antibody biodistribution 24 h after treatment. Using chimeric D31N as a positive control, we were able to confirm that all three humanized variants retain the important property of *in vivo* tumor localization (Figure 3E, 3F). In addition, we find that V66 demonstrates the highest tumor penetration signal, followed by V13, and then V31, which, markedly, is the order of decreasing nucleic acid affinity. This finding is in alignment with prior work demonstrating that cellular penetration *in vitro* is correlated with nucleic acid affinity [30]. Notably, this result marks the first time we have observed this trend *in vivo* for tumor localization. Taken together, the data in this figure demonstrate the exciting potential of these humanized 3E10 variants as therapeutic antibodies with a distinct mechanism of uptake suited for high efficiency tumor targeting. Additionally, the finding that the humanized variants’ tumor accumulation is related to nucleic acid affinity provides the basis for the rational advancement of select variants for optimal tumor targeting.

### Low nucleic acid affinity variants show superior binding to RAD51 and are synthetically lethal to BRCA2- and PTEN-deficient cells

**Figure 4:**
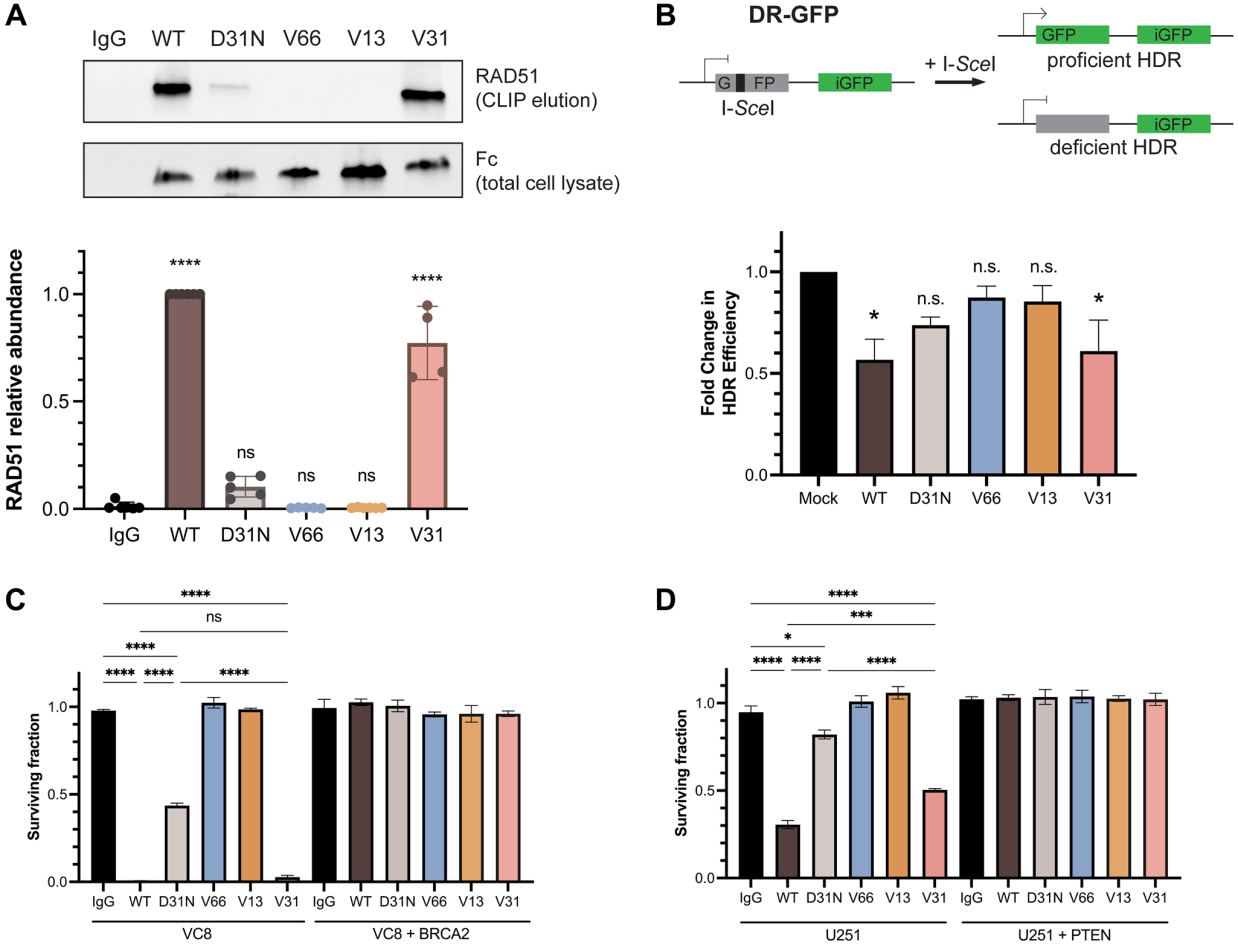
RAD51 binding and homology-directed repair inhibition properties are retained in a fully humanized 3E10 variant. (**A**) Representative western blot (top) showing RAD51 binding by antibodies as assessed following CLIP. MCF7 cells were transfected with 10 μg CMV-hRAD51 plasmid and treated with 500 nM antibodies prior to UV crosslinking and immunoprecipitation. Quantification (bottom) is of *n* = 4 replicates, where data are mean ± SEM. RAD51 abundance is first normalized to Fc (3E10 antibody) abundance to account for differences in antibody cellular penetration, and these values are then normalized to WT. (**B**) Quantification of homology-directed repair (HDR) in U2OS DR-GFP cells containing an inducible GFP reporter. Cells were treated with 1.2 μM antibody for 16–24 h and GFP expression was measured by flow cytometry. Data are mean ± SEM, *n* = 3 replicates. (**C**, **D**) Survival assays in isogenic cell lines. (C) VC8 ± BRCA2, and (D) U251 ± PTEN. VC8 cells were treated with 500 nM antibodies and U251 cells were treated with 1 μM antibodies for 4–5 days. Cell survival was measured using CellTiter-Glo ATP-based viability assay. Data are mean ± SEM, *n* = 3 replicates.

Previous reports have demonstrated that chimeric 3E10 binds to the N-terminal domain of RAD51 and, via inhibition of the HDR pathway, is synthetically lethal to BRCA2-deficient and PTEN-deficient cells *in vitro* [21, 30, 33]. Abnormalities in DNA repair and genomic instability, including HDR deficiency, represent a key hallmark of cancer and constitute one of the most common drivers of tumorigenesis [45]; thus, understanding the 3E10-RAD51 interaction is imperative for the advancement of this mAb toward the most effective therapy for the large cohort of patients with DNA-repair deficient tumors. We thus wanted to assess RAD51 binding and HDR inhibition in our panel of humanized 3E10 variants. Since prior work has shown that the lower affinity chimeric WT 3E10 has a higher affinity interaction with purified RAD51 [30], we hypothesized that, like cellular penetration and tumor localization, nucleic acid affinity could be predictive of RAD51 binding efficiency. To test this, we first assayed RAD51 binding by treating cells in culture with humanized antibodies and performing cross-linking and immunoprecipitation (CLIP) and western blot analysis of RAD51 abundance. Using WT and D31N chimeric 3E10 as controls, we find that out of the three humanized variants tested, only the low affinity antibody, V31, demonstrates RAD51 binding (Figure 4A).

To test the biological relevance of this observation, we assessed the effects of the humanized 3E10 antibodies on HDR efficiency using a cell-based direct repeat (DR)-GFP reporter assay, as previously described [46, 47]. Briefly, cells were first treated with 3E10 antibodies, followed by transfection of an I-*Sce*I plasmid which creates a site-specific double-strand break in one of two repeated GFP sequences. In the case of proficient HDR, the double-strand break is repaired and GFP expression is activated. Using this assay, we find that cells treated with WT and V31 antibodies show a significant decrease in the percentage of HDR-proficient cells, in line with the conclusions of our CLIP assay (Figure 4B). We also observe a slight (but statistically insignificant) decrease in HDR following D31N treatment, which is also in accordance with previous findings [30].

We then asked whether we could recapitulate the previously observed synthetic lethality in BRCA2- and PTEN-deficient cells using the humanized 3E10 variants. We first treated isogenic VC8 Chinese hamster cells (either BRCA2-expressing or BRCA2-null) with chimeric and humanized 3E10 variants and assessed survival using the CellTiter-Glo ATP-based viability assay. In accordance with our RAD51 CLIP and HDR inhibition assays, we find that some 3E10 variants are synthetically lethal in BRCA2-null (but not BRCA2-proficient) VC8 cells. Treatment with WT 3E10 and V31 lead to the largest decreases in BRCA2-null cell viability, followed by D31N (Figure 4C). No cell death is observed following treatment with V66 or V13, which also do not show evidence of RAD51 binding or HDR inhibition. We then repeated this same assay in a second isogenic cell line, U251 glioblastoma cells that are either PTEN-expressing or PTEN-null. We again find that the largest decrease in PTEN-null cell viability occurs following treatment with WT 3E10, followed by V31, and then D31N, all in agreement with prior data (Figure 4D).

**Figure 5:**
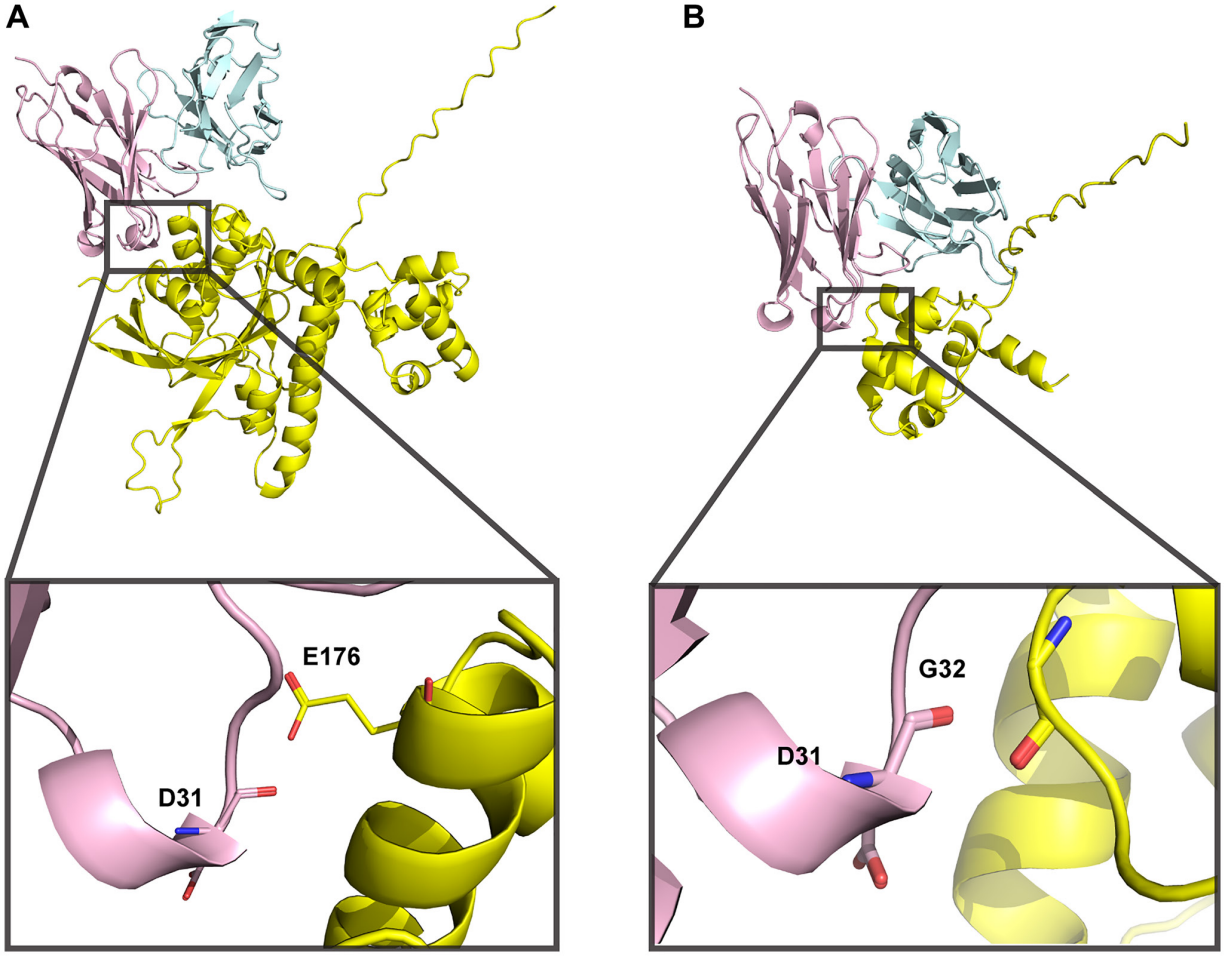
3E10-RAD51 interaction models generated using AlphaFold 3 are of low confidence. (**A**) Full-length human RAD51 sequence was used to predict docking to WT 3E10. iPTM = 0.42, pTM = 0.53. (**B**) N-terminal domain RAD51 sequence (aa 1–89) was used to predict docking to WT 3E10. iPTM = 0.6, pTM = 0.72. 3E10 heavy chains are shown in pink, light chains are shown in blue, and RAD51 is shown in yellow. Insets show RAD51 residues within close proximity to D31.

The data showing that V31, the variant with low nucleic acid affinity, exhibits RAD51 binding, HDR inhibition, and synthetic lethality with BRCA2- and PTEN-deficient cells led us to hypothesize that 3E10 RAD51 binding may also be related to differential nucleic acid affinity. To begin to ask this question, we explored models of the predicted protein-protein interaction (PPI). We attempted to use AlphaFold 3, which has significantly improved algorithms for protein-ligand and antibody-antigen binding compared to other platforms [48], to predict this interaction *de novo*. Based on prior work demonstrating that 3E10 binds to the N-terminal domain of RAD51 [30], we used both full-length and truncated N-terminal human RAD51 sequences as inputs. We find that these two models both predict the D31 residue in heavy chain CDR1 as being within interaction distance of the RAD51 interface (Figure 5A, 5B). However, we notably see that the predicted RAD51 binding surface is inconsistent between the full-length and N-terminal domain models; two entirely different RAD51 residues are in proximity to D31 for each model. Moreover, AlphaFold 3 generates rather low confidence scores for these models (iPTM = 0.42 and 0.6 for full-length RAD51 and N-terminal domain, respectively), leading us to conclude that experimental structural analysis will be necessary to uncover the details of the 3E10-RAD51 interaction. Nonetheless, experimental data assessing WT and D31N does demonstrate that the substitution of asparagine for aspartic acid at this position greatly decreases RAD51 binding. Furthermore, RAD51 binding increases when human 3E10 variant nucleic acid affinity decreases. It is therefore likely that RAD51 binds in a region similar to or the same as the 3E10 nucleic acid binding pocket (and may even experience competitive binding with nucleic acid ligands). However, rigorous structural studies currently being conducted in the lab will be of vital importance to interrogate the specific determinants of this interaction.

## DISCUSSION

The cell penetrating monoclonal antibody 3E10 has demonstrated strong therapeutic potential for tumor targeting in pre-clinical studies [20, 21, 30, 33]. It is therefore important to humanize the mAb for eventual use in patients. Data presented here demonstrate that multiple humanized 3E10 variants retain important biological properties and furthermore that these properties can be rationally modulated to fit oncologic indications of interest. Via *in silico* modeling and quantitative binding assays we identify 3E10’s nucleic acid binding pocket and demonstrate that changes in charge which can be predicted via modeling lead to widely variable affinities for nucleic acid ligands. We show that lower affinity nucleic acid binding is correlated with faster intracellular release of an mRNA ligand *in vitro*. We also show that the uptake of all humanized 3E10 variants is dependent on the ENT2 nucleoside transporter and bypasses endocytosis pathways. However, higher affinity nucleic acid binding correlates with more robust and faster cell uptake *in vitro*. Furthermore, all variants retain the ability to localize to tumors *in vivo*, with the most potent localization seen with the high affinity variant. Finally, we observe a trend between nucleic acid affinity and RAD51 binding and downstream HDR inhibition, and demonstrate that the lowest affinity humanized variant retains its synthetic lethality in BRCA2- and PTEN-deficient cells.

There is substantial clinical interest in therapeutic tumor delivery vehicles that can avoid payload off-target tissue uptake. Recently, monoclonal antibody-based therapies have made great strides in surpassing this limitation by harnessing surface antigens which are either overexpressed or uniquely expressed in tumors [1, 38]. However, challenges remain in the mAb therapy space, including the need to identify sufficiently unique and robustly expressed tumor antigens, exploit new mechanisms of tumor targeting, and overcome eventual drug resistance [4, 6]. Our data present humanized 3E10 as a novel modality to achieve superior tumor targeting and highly effective intracellular antibody delivery in an antigen-independent manner. This is demonstrated by robust 3E10 cellular penetration, potent mRNA delivery, and highly specific biodistribution to tumors.

Endocytic antibody uptake is considered one of the main challenges of mAb therapies, as it results in a general inability to reach intracellular targets and deliver intracellular payloads in the cytosol and especially in the nucleus. Entrapment of antibodies and their payloads in endosomes leads to significant lysosomal degradation, imposing pharmacokinetic challenges when dosing patients [49]. We notably show that humanized 3E10 cellular penetration is independent of endocytic cell uptake pathways and is instead highly dependent on the function of the ENT2 nucleoside transporter. ENT2 is present on both plasma and nuclear cell membranes [29] and therefore likely facilities the transduction of 3E10 past both barriers. ENT2 is also highly overexpressed in multiple human cancers due to high tumor cell proliferative and metabolic needs [26, 28]. This unique uptake pathway is therefore a preeminent determinant of two important clinical properties of 3E10: its cellular and nuclear penetration; and its tumor-specific localization. Though the exact mechanism of 3E10-ENT2 engagement is unknown and is a significant area of active investigation, our findings distinguish humanized 3E10 variants from other humanized mAbs and indicate their potential to overcome the limitations of currently available therapies.

An example of a class of intracellular targets that are yet unreachable with clinically available mAbs are DNA damage repair (DDR) proteins. Many of the most common genetic oncologic drivers are in genes which play a role in one (or more) of a myriad of DDR pathways [45]. With the logic that moderate genome instability and DNA repair deficiency may be increased to push cancer cells past the point of viability, many canonical chemotherapies function by inducing further DNA damage or inhibiting additional DDR proteins. Small-molecule chemotherapeutics are unfortunately often associated with severe side effects due to off-target tissue toxicity. Data presented here advance 3E10 as a unique inhibitor of a critical DDR protein, RAD51, which can also be used as a highly tumor-specific agent. We show that, of the humanized variants, V31 exhibits the most potent RAD51 binding, direct HDR inhibition, and synthetic lethality in DDR-deficient cells. Mechanistically, it is probable that the double hit to PTEN/BRCA2 (in cell lines which are deficient in one of these) and RAD51 (inhibited by V31) leads to replication fork stress, deprotection, and eventual collapse. Moreover, accumulated evidence suggests this effect of V31 and other 3E10 antibodies is associated specifically with RAD51 binding and consequent HDR inhibition, since prior work indicates that 3E10 antibodies do not affect the non-homologous end joining pathway [30]. NHEJ is the main alternative repair pathway to address DSBs that might arise from replication stress or of from other forms of DNA damage.

Unveiling the precise PPI domains and/or residues via rigorous structural assessment is an area of active research. Nonetheless, our data paves a path for the rational development of a precise humanized 3E10 HDR-specific inhibitor with further improved RAD51 binding, potentially via point substitutions or more global alterations to the electrostatic charge of the interaction region.

Overall, the data presented in this study affirm that humanizing 3E10 preserves its crucial biological properties essential for therapeutic efficacy. Moreover, our findings unveil a functional model that elucidates the intricate relationships between nucleic acid binding and delivery, antibody cellular penetration, tumor-specific localization, and RAD51 binding and subsequent synthetic lethality. Importantly, we demonstrate the range of mutability of these properties, offering a foundation for the rational design of new humanized 3E10 antibodies with diverse biomedical applications in areas of significant unmet clinical need. These encompass tumor-specific gene therapy delivery and intracellular antibody-based RAD51 targeting, among others. Given the variety of necessary drug properties between diseases, we herein establish a versatile therapeutic approach that is potentially adaptable to multiple clinical oncology indications, balancing considerations such as nucleic acid release kinetics, RAD51 affinity, uptake kinetics, and tumor localization. Taken together, these data underscore the potential of this antibody-based platform for precise tumor targeting following systemic administration and emphasize the importance of its continued research and development.

## MATERIALS AND METHODS

### Cell lines

U2OS DR-GFP cells were obtained from Dr. Ranjit Bindra (Yale University) and cultured in Dulbecco’s modified Eagle medium (DMEM) with 10% fetal bovine serum (FBS) and 1% penicillin/streptomycin (Pen/Strep). U251 and VC8 cells have been described previously [50, 51] and were cultured in DMEM with 10% FBS and 1% Pen/Strep. K562 (CCL-243, American Type Culture Collection (ATCC)) and MCF7 cells (HTB-22, ATCC) were maintained in RPMI-1640 medium with 10% FBS and 1% Pen/Strep. EMT6 (CRL-2755, ATCC) and HeLa (CRM-CCL-2, ATCC) cells were maintained in DMEM with 10% FBS and 1% Pen/Strep. All cell lines were tested and confirmed to be free of mycoplasma infection.

### 3E10 antibody humanization design and production

For humanization design, two human germlines (heavy chain: IGHV3-48*01 (84.7%) and light chain: IGKV7-3*01 (73.7%)) with highest homology to the mouse sequence were selected using IgBlast. Potential key residues were identified via modeling. 7 humanized variable heavy chains and 6 humanized variable light chains were thus generated. Of these, 22 full-length human 3E10 variants were generated from different permutations of these chains. Variants were designed, synthesized, expressed in Chinese hamster ovary cells, and purified following standard operating procedures (Genscript, Piscataway NJ). Purity was determined to be ≥90% by SDS-PAGE.

### ELISA – EC_50_ of purified humanized 3E10 antibodies

The affinity of purified antibodies to poly(dT) DNA was determined by ELISA. Poly(dT) ligand was immobilized on streptavidin pre-coated 96-well plates and incubated for 30 min at room temperature. Plates were washed with 1X TBST (20 mM Tris pH 7.4, 150 mM NaCl, 0.1% Tween^®^ 20 detergent) before serial dilutions of 3E10 antibody were added for 4 h at 4°C, followed by a second wash step and then incubation with goat anti-human Fc HRP secondary antibody (Invitrogen #31413) at room temperature for 90 min. Plates were washed with TBST and chemiluminescent substrate was added to wells and developed in the dark for 15 min. Finally, 1 M HCl buffer was added to each well to stop the reaction, and luminescence was determined on a Synergy H1 Multi-Mode Microplate Reader (Biotek).

### *In silico* modeling of DNA binding antibodies

Predicted structures of 3E10 variant scFvs were determined using IgFold (Version 0.0) hosted on the Cosmic2 virtual server (San Diego Supercomputer Center). Interaction models between 3E10 and poly(dT) were generated using AutoDock Vina (Version 1.2.5), and models between 3E10 and RAD51 were generated using AlphaFold (Version 3). Electrostatic maps were generated using the Adaptive Poisson-Boltzmann Solver (Version 3.4.1) and images were rendered using PyMOL Molecular Graphics System (Version 2.5.5).

### Affinity and complex stability assays

For biolayer interferometry, biotinylated poly(dT) was captured on streptavidin biosensors at 30 nM for 90 seconds. Antibodies were titrated from 100 nM to 1 nM in 1X PBST, pH 6.3. Kinetic measurement is representative of a 1:1 interaction model. Measurements are double referenced. On-rates (k_a_) were measured for 5 min, and off-rates (k_d_) were measured for 10 min on an Octet R8 Protein Analysis System (Sartorius). For zeta potential measurement, antibodies alone or antibody/poly(dT) complexes at a 5:1 molar ratio were diluted in water and charge was assessed using a Zetasizer Nano ZS (Malvern).

### ELISA – antibody nucleic acid binding

3’ biotinylated nucleic acids were diluted to 1 nM in 50 mM HEPES-KOH pH 7.9, 150 mM NaCl, 10 mM MgCl_2_, 0.7% Triton X-100, and 5% glycerol. 100 μL of this solution was added to a streptavidin pre-coated opaque white 96-well plate and incubated for 30 min at room temperature with rocking. Three washes with 1X TBST were performed before incubation with 100 μL antibodies at 33 ng/mL for 2 h at room temperature with rocking. Another three washes with 1X TBST were performed prior to the addition of goat anti-human Fc HRP secondary antibody (Invitrogen #31413) at a 1:20,000 dilution for 90 min at room temperature with rocking. Finally, plates were washed three more times with 1X TBST, developed with SuperSignal^™^ ELISA Pico Chemiluminescent Substrate (Thermo Scientific), and read in a Synergy H1 Multi-Mode Microplate Reader (Biotek).

### Flow cytometry – determination of GFP mRNA delivery

K562 cells were seeded in 12-well plates and treated with 3E10/GFP mRNA. Complexes were generated by mixing 3E10 (250 μg) and cytosolic GFP mRNA (10 μg, Genscript) and incubating at room temperature for 10 min. IgG1/mRNA mixtures were used as a negative control. Cells were incubated with complexes for 1–3 days and then fixed in 1% PFA at room temperature for 15 min. Samples were centrifuged at 300 × g for 5 min, washed with 1X phosphate-buffered saline (PBS), centrifuged again, resuspended in 300 μL 1X PBS, and passed through a 0.2 μm cell strainer. Samples were analyzed for GFP fluorescence using a CytoFLEX LX Flow Cytometer (Beckman Coulter). Gating and quantification were performed on CytoBank (Beckman Coulter).

### Immunofluorescence staining – cell penetration of 3E10 variants

HeLa cells were plated in 8-well glass chamber slides (MilliporeSigma) at 20,000 cells per well. Following treatment for 24 h, cells were washed once with PBS and fixed using solution containing 3% paraformaldehyde (Santa Cruz Biotechnology), 0.5% Triton X-100, and 8% sucrose (both MilliporeSigma) in PBS for 15 min at room temperature. Subsequently, cells were washed twice with PBS and incubated with blocking solution containing 5% Normal Goat Serum (Invitrogen), 0.5% Triton X-100, and 8% sucrose in PBS overnight at 4°C. Next, samples were incubated with 1:400 goat anti-human FITC-conjugated secondary antibody (Jackson Immuno #109-545-170) and 1:400 Alexa Fluor 594-conjugated phalloidin (Invitrogen) in blocking solution for 120 min at room temperature, followed by 3x 5 min washes with PBS, with subsequent nuclei staining using 2 μg/mL DAPI (MilliporeSigma) in PBS solution for 15 min at room temperature. After 3x final 5 min washes with PBS, slides were sealed using ProLong Glass Antifade media (Invitrogen) and #1.5 glass cover slips (Corning), then stored in −20°C and protected from light until analyzed.

### Fluorescence image acquisition and analysis

Fluorescence images were acquired on the Leica Stellaris 8 Falcon laser scanning confocal microscope using the HC PL APO 63x/1,40 OIL objective with Type F Immersion liquid (Leica Microsystems). 405 nm laser was used for DAPI and White Light Laser (WLL) was used for FITC and Alexa Fluor 594 fluorophore excitation with subsequent detection by HyD detectors. Sequential scanning was optimized for signal yields and to prevent fluorescence crosstalk by the Leica Application Suit X (LAS X) software. All images were taken with three quarters of the maximum intensity without overexposure and all exposure, laser intensity, and gain parameters were kept constant for all samples. The pictures were saved as 1024 pixels × 1024 pixels, 8-bit multi-channel Leica Image Files (.lif) with no further editing. For fluorescence intensities quantification, images were additionally exported to 8-bit TIFF format files and were quantified using Focinator software [52]. Data are ≥250 cells per group, and statistical significance was determined using a non-parametric Kruskal-Wallis test with multiple comparisons.

### Flow cytometry – cell penetration of 3E10 variants

3E10 antibodies were directly labeled with IVISense 680 NHS Fluorescent Dye (Revvity) in 50 mM carbonate/bicarbonate buffer, pH 8.5 for 2 h at room temperature and purified using Zeba spin desalting columns. K562 cells were pre-treated with inhibitors for 30 min, then treated with inhibitors and 750 nM AlexaFluor 680-labeled antibodies at 37°C, 5% CO_2_ for 1 h. Following treatment, cells were washed twice with PBS, trypsinized, and centrifuged at 300 × g for 5 min prior to preparation for flow cytometry as described above. Chemical inhibitors were obtained from the following sources and used at the following concentrations: bafilomycin A1 (Sigma Aldrich), 500 nM; chlorpromazine HCl (Cayman Chemical), 5 μg/mL; dipyridamole (Sigma Aldrich), 50 μM; 5-(N-ethyl-N-isopropyl)-amiloride (Cayman Chemical), 25 μM; filipin III (Cayman Chemical), 1 μg/mL; *S*-(4-nitrobenzyl)-6-thioinosine (Millipore Sigma), 100 μM. Three biological replicates were performed, and statistical significance was determined using two-way ANOVA.

### Biodistribution in EMT6 tumors

All mouse studies were approved by the Yale University Institutional Animal Care and Use Committee. In all cases, female mice 6 to 8 weeks of age were used and kept in temperature-controlled environments with 12-h light cycles and free access to water and food. For EMT6 breast cancer tumors, 1E6 cells were implanted subcutaneously in 100 μL of media in the right flanks of BALB/c mice. Mice were treated with 100 μg IVISense 680 NHS Fluorescent Dye (Revvity) labeled antibodies once tumor volumes reached 150–200 mm^3^. Mice were anesthetized with 2.5% isoflurane prior to treatments, and antibodies were administered by intravenous retro-orbital injection. After 24 h, tumors and major organs were harvested and imaged using the IVIS Spectrum *In Vivo* Imaging System (Perkin Elmer).

### Cross-link and immunoprecipitation and western blot analysis of 3E10-RAD51 interaction

MCF7 cells were grown to 65% confluency in 10 cm dishes and transiently transfected with 10 μg CMV-hRAD51 plasmid (Addgene #125570) using FuGENE 4K (Promega). The next day, cells were treated with 500 nM of purified antibodies for 6 h. Cells were UV crosslinked prior to cellular lysate harvesting. Cells were lysed using Pierce co-IP Lysis/Wash Buffer as described in the manufacturer’s protocol (Thermo Scientific), and the protein concentration of each sample was determined using the DC^™^ protein assay. Protein A/G beads were washed according to the manufacturer’s protocol, and 500 μg of cell lysates were incubated with the beads using over-end mixing at room temperature for 2 h. Complexes were eluted in low-pH buffer and neutralized according to the manufacturer’s protocol. Eluates and whole cell lysates were run on 4–20% gradient SDS-PAGE gels (Bio-Rad StainFree TGX) and transferred for western blotting. The primary antibodies used were rabbit anti-RAD51 (Cell Signaling Technology #8875S) for the eluate blot and HRP-conjugated goat anti-human Fc (Invitrogen #31413) for the whole lysate blot. These antibodies were diluted in 5% milk at 1:1000 and 1:10,000, respectively, and incubated for 1–3 h at room temperature. Secondary goat anti-rabbit antibody (Invitrogen #31460) was used at 1:10,000 in 5% milk and incubated for 1 h at room temperature. Two washes with TBST were performed after primary and secondary antibody incubations, and membranes were developed using Clarity Max^™^ Western ECL Substrate (Bio-Rad). Fc total lysate signals were quantified and normalized to stain-free gel total protein using Image Lab 6.1 (Bio-Rad). RAD51 signals were then normalized to Fc total lysate signals. Five biological replicates were performed, and statistical significance was determined using Student’s two-tailed paired *t*-test.

### Flow cytometry – U2OS DR-GFP reporter assay

The DR-GFP assay was performed using U2OS DR-GFP cells as previously described [46, 47]. Briefly, cells were seeded and pretreated with 1.2 μM antibody for 16–24 h. Site-specific double-strand breaks were introduced using the Amaxa Nucleofector II and Nucleofector Kit V (Lonza) to deliver 4 μg plasmid encoding the restriction enzyme I-SceI. After 72 h, cells were analyzed by flow cytometry to quantify GFP-positive cells as described above. Three biological replicates were performed, and statistical significance was determined using a one-way ANOVA with multiple comparisons.

### Cell viability assays

Cells were seeded at 500 cells per well in a 96-well plate and treated with purified 3E10 antibodies. Five days after treatment, viability was assessed using the CellTiter-Glo Luminescent Cell Viability Assay (Promega) according to the manufacturer’s protocol. Three biological replicates were performed, and statistical significance was determined using a one-way ANOVA with multiple comparisons.

### Western analysis of ENT2 expression

Whole cell lysates were run on 4–20% gradient SDS-PAGE gels (Bio-Rad StainFree TGX) and transferred for western blotting. The primary antibodies used were rabbit anti-ENT2 (Abcam #181192) and HRP-conjugated mouse anti-GAPDH (Proteintech #60004). These antibodies were diluted in 5% milk at 1:1000 and 1:10,000, respectively, and incubated for 1 h at room temperature. Secondary goat anti-rabbit antibody (Invitrogen #31460) was used at 1:10,000 in 5% milk and incubated for 1 h at room temperature. Two washes with TBST were performed after primary and secondary antibody incubations, and membranes were developed using Clarity Max^™^ Western ECL Substrate (Bio-Rad).

## SUPPLEMENTARY MATERIALS



## Abbreviations

ATCC: American Type Culture Collection
BLI: biolayer interferometry
CDR: complementarity determining region
CLIP: cross-linking and immunoprecipitation
DDR: DNA damage repair
DMEM: Dulbecco’s modified Eagle medium
DR: direct repeat
FBS: fetal bovine serum
HDR: homology-directed repair
mAb: monoclonal antibody
PBS: phosphate-buffered saline
PPI: protein-protein interaction
scFv: single-chain variable fragment
SLE: systemic lupus erythematosus
