# Supplementary material for: Next-generation cell-penetrating antibodies for tumor targeting and RAD51 inhibition
Source: Oncotarget. 2024 Oct 1;15:699–713. doi: 10.18632/oncotarget.28651 (PMC11444335; doi:10.18632/oncotarget.28651)
Supplement: Supplementary file 1 [file oncotarget-15-28651-s001.pdf]

Next-generation cell-penetrating antibodies for tumor targeting and RAD51 inhibition

SUPPLEMENTARY MATERIALS

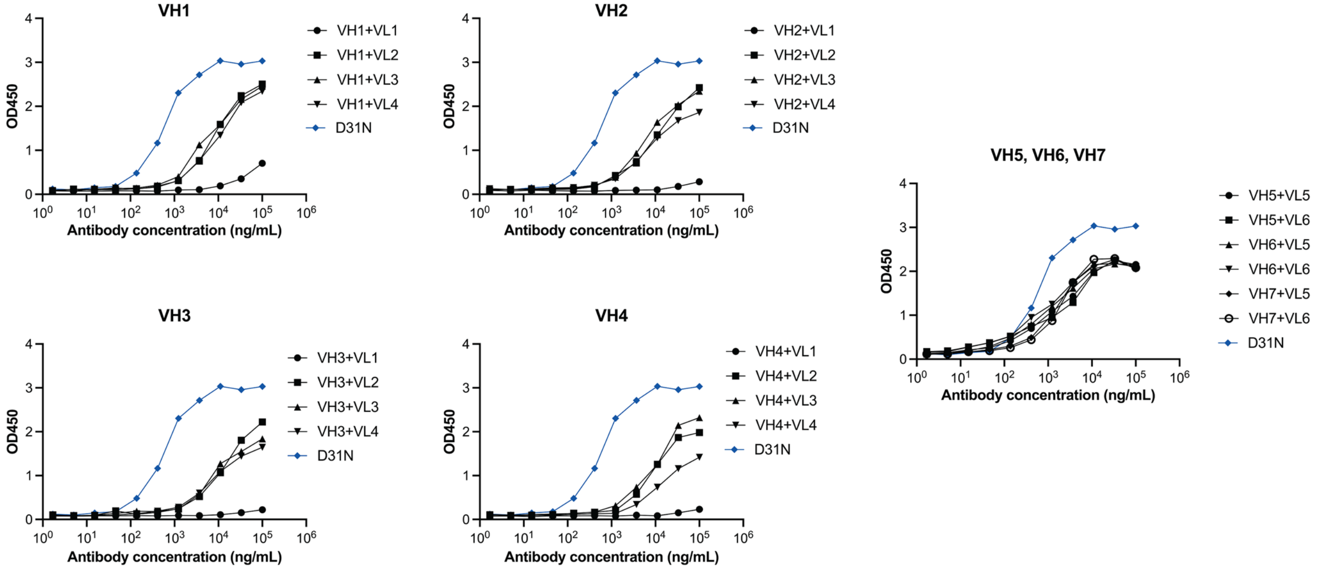

Supplementary Figure 1: ELISA screening of 22 humanized 3E10 variants for poly(dT) EC_{50}. Plots demonstrating the antibody/DNA binding curves from which EC_{50}s for all variants were mathematically derived. In each experiment, D31N was used as a positive control.

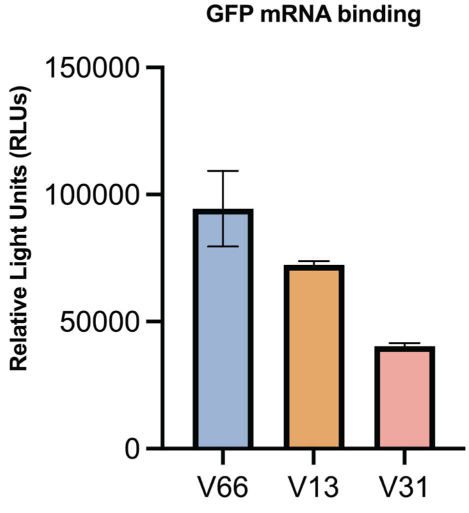

Supplementary Figure 2: Determination of 3E10 variant GFP mRNA binding affinity. ELISA assay measuring V66, V13, and V31 affinity to 3′-biotinylated mRNA encoding cytosolic GFP. Data are mean ± SEM, $n = 3$ replicates.

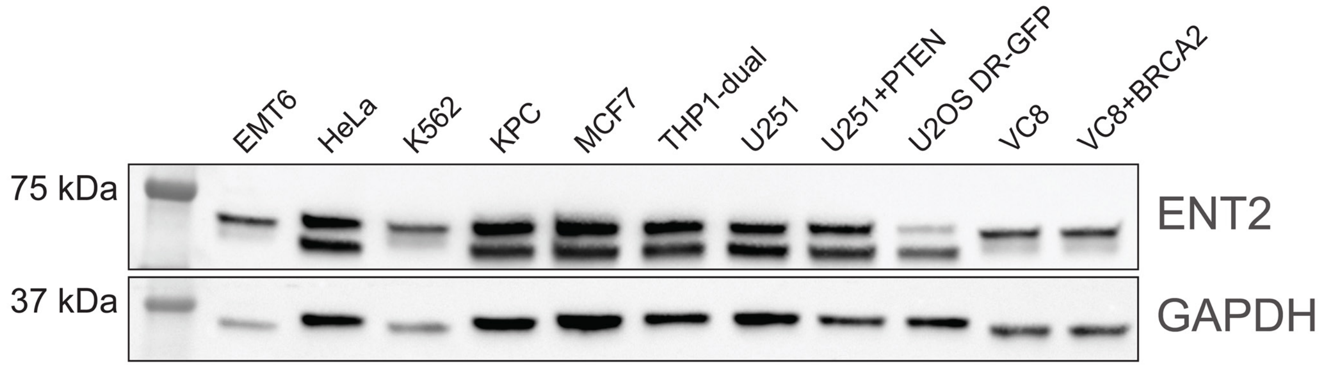

Supplementary Figure 3: Determination of ENT2 expression in human cell lines. ENT2 (~60 and ~55 kDa bands) relative abundance in all cell lines used for 3E10 antibody and/or nucleic acid payload uptake was assessed using western blot analysis.
